# Assessing the Impact of Vegan Toothpastes on Enamel Color Change and Surface Properties

**DOI:** 10.1111/jerd.13483

**Published:** 2025-05-13

**Authors:** Reginna Vyctoria da Trindade Souza de Melo Carneiro, Raissa Manoel Garcia, Tais Scaramucci, Vanessa Cavalli

**Affiliations:** ^1^ Department of Restorative Dentistry Piracicaba Dental School, University of Campinas São Paulo Brazil; ^2^ Department of Restorative Dentistry School of Dentistry, University of São Paulo São Paulo Brazil

**Keywords:** dental enamel, tooth abrasion, toothbrushing, toothpastes, vegans

## Abstract

**Objective:**

To evaluate color (ΔE_00_) and whiteness index (ΔWI_D_) changes, surface hardness loss (SHL), roughness (Ra), mineral content, and morphology of enamel brushed with vegan toothpastes.

**Methods:**

Enamel blocks (*n* = 10/group) were stained with black tea (T0) underwent 15.000 (T1) and 30.000 (T2) toothbrushing cycles with: (*n* = 10): (AS) Artificial Saliva (control); (CT) Colgate Total 12 Clean Mint; (CC) Turmeric, Clove, Tea Tree Extracts; (CA) Chamomile, Melissa and Grape Extracts; (CH) Pepper Mint and Turmeric Extracts; (ZM) Zero Mint; (ZH) Zero Pepper Mint; (ME) Mint Everest; (CM) Charcoal and Mint; (HA) Herbal Anis Mint and Tea Tree. Color parameters (ΔE_00_, ΔWI_D_), SHL, and Ra were recorded at T0, T1, and T2. At T2, mineral content (EDS) and surface morphology (SEM) were examined. Data were statistically analyzed (α = 0.05).

**Results:**

At T1, no differences in ΔE_00_ and %SHL were observed among groups (*p* > 0.05). At T2, ZM, ZH, ME, and CM displayed higher ΔE_00_ than AS, CC exhibited the highest %SHL, and Ra increased for all groups (*p* < 0.05). At T1 and T2, all toothpastes caused negative ΔWI_D_ values. None of the toothpastes triggered changes in enamel mineral content or surface morphology.

**Conclusions:**

Vegan toothpastes did not cause hardness loss or deleterious effects on enamel mineral content or morphology. However, they exhibited no whitening effect and increased enamel roughness.

**Clinical Significance:**

Toothpastes indicated for vegan individuals are available, but there is little evidence to support their use, in contrast to their widespread marketing. Although vegan toothpastes did not change enamel microhardness nor promote adverse effects on enamel morphology, none of the toothpastes tested were able to exhibit a potential whitening effect.

## 1 Introduction

Adopting vegetarian habits in various forms has become popular in recent years. Among the different forms of vegetarian diets, the main difference between them is the exclusion of specific food categories. Vegan diets adopt a vegetarian habit in which the individual does not consume foods of animal origin and their by‐products, thus being a stricter diet [1]. Further categories of vegetarians include lacto‐vegetarians (in which meat, fish, and eggs are excluded, but dairy products are allowed), ovo‐vegetarians (in which meat, fish, and dairy products are excluded, but eggs consumption is allowed), lacto‐ovo vegetarians (in which meat and fish are banned but eggs and dairy products are allowed), and pescatarians (restricting meat consumption to fish and seafood only) [1, 2].

Because of the restrictions of these diets, manufacturers were inclined to develop various innovative oral‐care products, including toothbrushes, toothpastes, dental floss, and mouthwashes, specifically designed and recommended for vegetarians or vegan individuals. Regardless of their claim or specific “natural” composition, these products must effectively clean dental surfaces, thus contributing to oral health maintenance. With that in mind, the effects of commercial toothpastes and mouth rinses containing natural/herbal agents on biofilm viability, extracellular polysaccharide (EPS) production, and enamel demineralization in vitro have been recently evaluated [3]. That report demonstrated that some toothpastes and mouth rinses significantly reduced biofilm viability, but only one mouth rinse significantly influenced biofilm thickness and EPS. Besides, the authors showed that the presence of natural agents displayed different antimicrobial effects depending on their composition. Nevertheless, only some of them reduced mineral loss and exhibited anticaries effects.

The manufacturers of vegan toothpastes indicate that these oral hygiene products are formulated without animal‐derived ingredients or animal testing, making them an ethical and sustainable option for people who follow a vegan philosophy or seek to reduce the environmental impact of their consumption habits. In addition to not containing ingredients of animal origin, they also avoid components commonly used in traditional products. Ingredients commonly found in vegan toothpastes are vegetable glycerin, calcium carbonate, silica, sodium bicarbonate, essential oils, and xylitol.

Some of these oral care products claim to provide “natural teeth whitening.” Despite their “ecologically friendly” appeal and lack of fluorides and animal‐derived ingredients, some components like turmeric and activated charcoal correlate to “natural whitening.” Many patients look for toothpastes with a whitening appeal, and there are several commercially available toothpastes to avoid undergoing home and in‐office bleaching treatments based on hydrogen and carbamide peroxide. Nonetheless, professionals and patients must be mindful of the potential abrasiveness of these products, which may be “natural” but composed of abrasive particles [4, 5]. In a previous report, the effects of natural whitening products on color change and enamel surface properties were accessed [6]. The results demonstrated that popular whitening products were inefficient for teeth whitening, contrary to the widespread marketing of these products.

Based on the exposed and because of the diversity of oral‐care products claiming to prevent enamel mineral loss and provide tooth whitening, it is essential to evaluate the effects of these products on dental enamel to predict further clinical implications. Therefore, in this study, we aimed to assess whether or not tooth brushing with vegan toothpaste changes the color, microhardness, surface roughness, mineral content, and morphology of the enamel. The null hypotheses tested were that vegan toothpastes after 30.000 simulated brushing cycles (1) would not promote a whitening effect; (2) would not cause enamel surface hardness loss; (3) would not increase enamel surface roughness. Besides, the effects of vegan toothpastes on mineral content and enamel surface were qualitatively assessed after treatments.

## 2 Materials and Methods

### 2.1 Study Design

Bovine enamel blocks (*n* = 10) were tested in a two‐factorial design study, with “toothpaste” (described in Table 1) and “time” (after staining with black tea—T0, after 15.000 brushing cycles—T1, and after 30.000 brushing cycles—T2) as study factors. As controls, artificial saliva (the same as used for slurry preparation) and a conventional fluoride toothpaste (Colgate Total 12 Clean Mint) were used. Color analysis (ΔE_00_, ΔWI_D_), percentage of surface hardness loss (%SHL) and surface roughness (roughness average—Ra) were evaluated after T0, T1, and T2, while the enamel mineral content (EDS) (*n* = 3) and morphology (SEM) (*n* = 3) were evaluated at T2.

**TABLE 1:** Description of experimental groups.

Group	Commercial name	Basic composition	Natural components	Abrasive particles	Fluoride	Manufacturer/Lot
AS	Brushing with artificial saliva	0.213 g/L CaCl_2_*2H_2_O; 0.738 g/L KH_2_PO_4_; 1.114 g/L KCl; 0.381 g/L NaCl and 12 g/L of Tris Buffer; pH adjusted to 7.0 with concentrated HCl solution.	—	—	—	—
CT	Colgate Total 12 Clean Mint	Water, glycerin, aroma, sodium lauryl sulfate, triclosan 0.3%, PVM/MA copolymer, sorbitol, carrageenan, saccharin sodium, sodium hydroxide, white dye CI 77891	—	Hydrated silica	Sodium fluoride (1450 ppm fluoride)	Colgate palmolive, São Bernardo do Campo, SP, Brazil/3009BR1226
CC	Turmeric, Clove and Tea tree Extracts (Extratos de Cúrcuma, Cravo e Melaleuca)	Water, glycerin, aroma, xylitol, stevioside, xanthan gum, lauryl glucoside, sodium benzoate	Turmeric, Callus extract, Eugenia caroyphyllus leaf extract, Melaleuca alternifolia flower/leaf/stem extract	Hydrated silica, calcium carbonate	—	Orgânico Natural, Uberlândia, MG, Brazil/C2008
CA	Chamomile, Melissa and Grape Extracts (Extratos de Camomila, Melissa e Uva)	Water, glycerin, aroma, xylitol, stevioside, xanthan gum, lauryl glucoside, sodium benzoate	Vitis vinífera extract, Chamomilla recucita, flower extract, *Melissa officinalis* leaf extract	Hydrated sílica, calcium carbonate	—	Orgânico Natural, Uberlândia, MG, Brazil/B2005
CH	Pepper Mint and Turmeric Extracts (Extratos de Hortelã e Cúrcuma)	Water, glycerin, aroma, xylitol, stevioside, xanthan gum, sodium lauroyl sarcosinate, benzyl alcohol, D‐Limonene	*Mentha arvensis* leaf oil, *Calendula officinalis* flower extract, menthol, *Citrus aurantifolia* oil, sucralose, *Citrus grandis* Peel oil, Melaleuca alternafolia leaf oil, *Curcuma longa* rhizome extract	Hydrated silica Calcium carbonate, sodium bicarbonate	—	Boni Natural, São Paulo, SP, Brazil/3642/22‐1
ZM	Zero Mint (Colgate Zero Menta)	Water, glycerin, aroma, xylitol, sodium lauryl sulfate, sorbitol, cellulose gum, PEG‐12, stevia rebaudiana extract, rebaudioside A, benzyl alcohol, limonene	Limonene	Hydrated silica	Sodium fluoride (1100 ppm fluoride)	Colgate Palmolive, São Bernardo do Campo, SP, Brazil/1174MX11H5
ZH	Colgate Zero Pepper Mint (Colgate Zero Hortelã)	Water, glycerin, aroma, xylitol, sodium lauryl sulfate, sorbitol, cellulose gum, PEG‐12, stevia rebaudiana extract, rebaudioside A, benzyl alcohol	Limonene	Hydrated silica	Sodium fluoride (1100 ppm fluoride)	Colgate Palmolive, São Bernardo do Campo, SP, Brazil/1202MX11H3
ME	Mint Everest (Menta Everest)	Water, glycerin, aroma, xylitol, sodium lauryl sulfate, sorbitol, cellulose gum, benzyl alcohol, eugenol, sodium saccharin	Cinnamal	Sodium silicate, calcium carbonate, tetrasodium pyrophosphate, disodium pyrophosphate	Sodium monofluorophosphate (1200 ppm fluoride)	Ultra Action, São Paulo, SP, Brazil/162
CM	Charcoal and Mint (Carvão Vegetal e Menta)	Water, glycerin, sodium lauroyl sarcosinate, xanthan gum	*Mentha arvensis* leaf oil, xylitol, benzyl alcohol, *Eucalyptus globulus* leaf oil, *Calendula officinalis* flower extract, menthol, *Citrus aurantifolia* oil, sucralose, Melaleuca alternafolia leaf oil, D‐Limonene	Hydrated silica calcium carbonate, sodium bicarbonate, activated charcoal	—	Boni Natural, São Paulo, SP, Brazil/4781/22‐1
HA	Mint and Tea Tree (Herbal Anis Menta e Melaleuca)	Water, aroma, glycerol, xantham gum, sodium laurylsulfate, sodium carmelose, benzyl alcohol, sodium saccharin, sodium hydroxide, CI 74160 (blue colorant 74 160)	Melaleuca alternifolia leaf oil, Limonene	Hydrated silica, calcium carbonate, sodium bicarbonate, tetrasodium pyrophosphate	Sodium monofluorophophaste (1450 ppm fluoride)	Colgate Palmolive, São Bernardo do Campo, SP, Brazil/3027BR122HE

*Note:* * is part of formulation in artificial saliva.

The sample size was initially calculated on a pilot study, with a power of 80%, a significance level of 5%, using ΔE_00_ means and standard deviation values of AS and CT groups, resulting in a requirement of four specimens per group. However, considering the number of specimens adopted in previous studies employing similar methodology [7, 8], 10 specimens per group were ultimately utilized.

### 2.2 Sample Preparation

One hundred bovine incisors were selected, cleaned with traditional dental prophylaxis, and disinfected in 0.1% thymol solution (Dinâmica, Indaiatuba, SP, Brazil). The crowns were sectioned from the root with a double‐sided diamond disc (KG Sorensen, São Paulo, SP, Brazil). After removing the roots, dental enamel blocks, measuring 5 × 5 × 3 mm, were obtained using a low‐speed diamond saw (Isomet, Buehler, Lake Bluff, IL, USA) under copious water irrigation from the middle portion of the crown. The dentin surface of the blocks was flattened using a polishing machine (Arotec, São Paulo, SP, Brazil) with silicon carbide (SiC) sandpaper #320 to allow parallelism with the buccal surface of the enamel. Subsequently, the enamel was flattened with SiC sandpaper #600, #1200, and #2000 and polished with a felt disc and a diamond suspension, with 1 μm abrasive particles for 1 min. The initial surface microhardness (SMH) was obtained by averaging three readings, with a distance of 100 μm between them, in the central region of the specimen, with a Knoop type indenter (Future Tech‐FM‐1e, Tokyo, Japan, load of 50 g for 5 s) [9]. The mean obtained from SMH and the selected specimens exhibited mean values of up to 10% (+/−) of the overall mean.

### 2.3 Enamel Artificial Staining Protocol

The enamel blocks' lateral and dentin surfaces were covered with a thin layer of nail polish colorless so that only the buccal enamel was exposed. The exposed surface of the enamel was immersed for 24 h, at room temperature, in a buffered solution of black tea (Dr. Oetker, São Paulo, SP, Brazil, pH = 7.0), prepared with 2 g of black tea diluted in 100 mL of distilled water for 5 min, following the study protocol of previous studies [10, 11]. Then, the samples were brushed with pumice powder to remove nonadherent particles and then kept in artificial saliva (1.5 mM Ca, 0.9 mM P, 150 mM KCl, and 0.1 M Tris, pH 7.0) [12] for 7 days (replaced every 2 days) at 37°C to stabilize the color. After stabilization, the initial color analysis was carried out with a digital spectrophotometer (Easyshade 4.0, Vita Zahnfabrik, Bad Säckingen, Germany) to randomize the samples into groups according to the L* coordinate.

### 2.4 Group Division

After staining with black tea, all samples were randomly divided into 10 groups (*n* = 10) as follows:

(AS) Artificial Saliva (control);
(CT) Colgate Total 12 Clean Mint (Colgate);
(CC) Turmeric, Clove and Tea Tree Extracts (Natural);
(CA) Chamomile, Melissa and Grape Extracts (Natural);
(CH) Pepper Mint and Turmeric Extracts (Boni Natural);
(ZM) Zero Mint (Colgate);
(ZH) Zero Pepper Mint (Colgate);
(ME) Mint Everest (Ultra Action);
(CM) Charcoal and Mint (Boni Natural);
(HA) Herbal Anis Mint and Tea Tree (Sorriso).

### 2.5 Simulated Mechanical Toothbrushing

To simulate the brushing process, the specimens were fixed in PVC matrices and positioned in a brushing simulator (3 TOP Plus, Odeme Dental Research, Luzerna, SC, Brazil), using slurries from the tested toothpastes (according to the group) and artificial saliva (1.5 mM Ca, 0.9 mM P, 150 mM KCl, and 0.1 M Tris, pH 7.0), in a 1:3 ratio (wt./wt.), prepared before the abrasive challenge. The abrasive challenge consisted of 15.000 and 30.000 cycles, which are equivalent to 18 and 36 months of brushing [13, 14, 15], using soft toothbrushes (Oral‐B Indicator 30, Procter & Gamble, Manaus, AM, Brazil) and a force of 1.5 N. All procedures were carried out at room temperature (~24°C). At the end of each cycle, the specimens were washed with distilled water and stored in individual pots containing 3 mL of artificial saliva for 24 h.

### 2.6 Colorimetric Evaluation

Color evaluation was performed as described by Matos et al. [11]. A digital spectrophotometer (Easyshade 4.0, Vita Zahnfabrik, Bad Säckingen, Germany) determined the color parameters L* (black‐white axis), a* (red‐green axis), and b* (yellow‐blue axis). To standardize the positioning of the device tip, the spectrophotometer was attached to a three‐fingered laboratory clamp with the tip pointing downwards. The specimens were placed over a white opaque ceramic background in a lifting platform (Jack lift—Q219, Quimis) to allow contact with the device tip. This set was placed in a color‐matching lightbox using standard daylight mode (GTI Minimatcher Series, GTI Graphic Technology Inc., Newburgh, NY, USA), and color measurements were performed on each specimen in different directions by rotating the specimen and the ceramic underneath, without moving the spectrophotometer [11].

Color change was evaluated using the CIEDE2000 formula:

$$\begin{array}{c} {\mathrm{\Delta E}}_{00}\;\left(T1-T0\;\text{and}\;T2-T0\right)=\left[{\left({\mathrm{\Delta L}}^{\prime }/K_{L}S_{L}\right)}^{2}+{\left({\mathrm{\Delta C}}^{\prime }/K_{C}S_{C}\right)}^{2}\right.\\ {\left.+{\left({\mathrm{\Delta H}}^{\prime }/K_{H}S_{H}\right)}^{2}+{\mathrm{RT}}^{*}{\left({\mathrm{\Delta C}}^{\prime }/K_{C}S_{C}\right)}^{*}\left({\mathrm{\Delta H}}^{\prime }/K_{H}S_{H}\right)\right]}^{1/2} \end{array}$$

The whiteness index for dentistry (ΔWI_D_) was calculated according to the equation:${\mathrm{WI}}_{D}=0.511\;L^{*}-2.324\;a^{*}-1.100\;b^{*}$ [11, 16, 17]. ΔE_00_ and ΔWI_D_ were determined at two time points (T1 − T0) and (T2 − T0). Color change values (ΔE_00_) adopted for perception (PT) and acceptance (AT) limits (50:50%) were 0.8 (PT) and 1.8 (AT) units. The ΔWI_D_ mean units adopted for PT and AT limits (50:50%) were 0.7 (PT) and 2.6 (AT) units [18].

### 2.7 Percentage of Surface Hardness Loss (%SHL)

The samples were evaluated using a microhardness tester (HMV‐2000, Shimadzu, Tokyo, Japan) equipped with a Knoop indenter. A load of 50 g was applied for 5 s [9]. Three indentations were made for each sample, with a standard 100 μm distance between them. The average of the three readings was calculated for each sample. Surface microhardness (SMH) was expressed as the percentage of surface hardness loss (%SHL), calculated using the following formula, adapted from a previous investigation [19]:

$$\%\mathrm{SHL}=\left(\mathrm{SH}\;\text{baseline}-\mathrm{SH}\;\text{after brushing cycle}/\mathrm{SH}\;\text{baseline}\;x\;100\right)$$

### 2.8 Surface Roughness

The surface roughness was evaluated using a surface profile measuring device (Mitutoyo SJ‐410, Mitutoyo, Jundiaí, SP, Brazil). Three measurements were performed on each sample in the horizontal, vertical, and oblique directions to calculate the average roughness (Ra) and promote a homogeneous reading of the entire sample. The analysis was performed under the following parameters: cut‐off of 0.25 mm, static load of 5 N, and speed of 0.5 mm/s [11].

### 2.9 Scanning Electron Microscope (SEM) and Energy Dispersive X‐Ray Spectroscopy (EDS)

Representative samples from each group (*n* = 3 to SEM and *n* = 3 to EDS/group) were selected and analyzed at T2 [11]. Specimens were washed in an ultrasonic bath (Ultra Cleaner, Unique, Indaiatuba, SP, Brazil) for 10 min and dried overnight in an oven at 37°C. After drying, specimens were sputter‐coated with a thin carbon layer and observed under SEM (JEOL‐JSM, 6460LV, Tokyo, Japan), operating at 15 kV in vacuum mode (45 Pa). Images were recorded at 1000× magnification. Alongside the SEM images acquisition, the software of the energy‐dispersive X‐ray spectroscopy (EDS, Vantage System—Easymicro Noran Instruments, Middleton, Wisconsin, USA) provided semi‐qualitative data on the percentage of chemical elements (atomic percentage) in three standard areas (600 μm^2^) of the specimens.

### 2.10 Statistical Analysis

Exploratory data analysis was carried out to determine normal distributions and homoscedasticity (Shapiro Wilk and Levene). ∆E_00_, which did not fit a normal distribution, was analyzed using Kruskal–Wallis and Dunn's nonparametric tests for comparison between groups, while ∆WI_D_ was analyzed using one‐way ANOVA and Tukey post‐test. Microhardness data were analyzed using a linear mixed model for repeated measures over time, examining the effects of group, time, and the interaction between them. Since surface roughness data did not meet the assumptions for normal distribution, a generalized linear mixed model for repeated measurements over time and the effects of group, time, and their interaction was used. The software R and SPSS 20.0 were used to carry out data analysis. A significance level of 5% was adopted in all analyses.

## 3 Results

### 3.1 Colorimetric Evaluation

**FIGURE 1:**
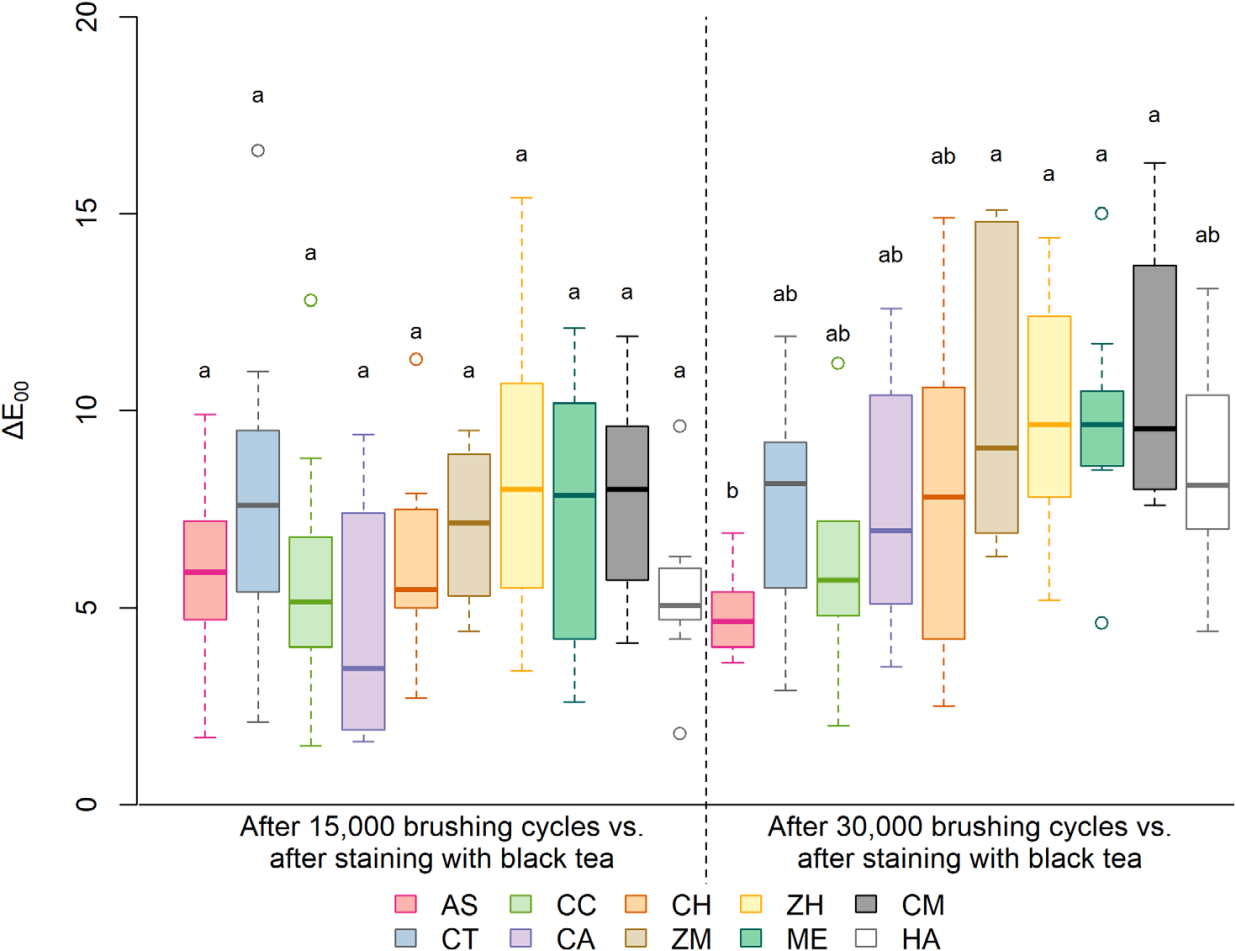
Mean values and standard deviations of ∆E_00_ at T1 and T2, respectively. Different letters indicate statistical differences between groups (*p* ≤ 0.05). AS, Artificial Saliva; CT, Colgate Total 12 Clean Mint; CC, Turmeric, Clove and Tea Tree Extracts; CA, Chamomile, Melissa and Grape Extracts; CH, Pepper Mint and Turmeric Extracts;ZM, Zero Mint; ZH, Zero Pepper Mint; ME, Mint Everest; CM, Charcoal and Mint; HA, Herbal Anis Mint and Tea Tree.

Figure 1 depicts T color change (∆E_00_) results at T1 and T2, respectively. Regardless of the evaluation time, all groups exhibited mean ΔE_00_ values above 0.8 and 1.8 units, the limits established for color perception and acceptability. At T1, no significant differences were noted among groups following 15.000 brushing cycles (*p* > 0.05). However, after T2, ZM, ZH, ME, and CM exhibited higher ∆E_00_ than the AS group (*p* < 0.05).

**FIGURE 2:**
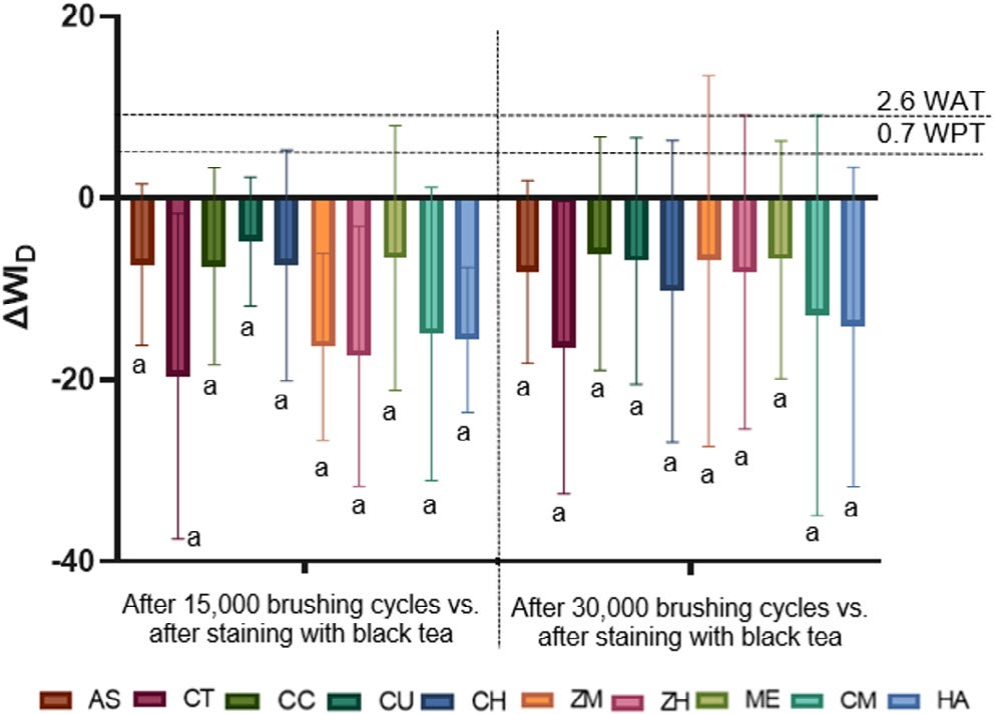
Mean values and standard deviations of ΔWI_D_ at T1 and T2, respectively. Different letters indicate statistical differences between groups (*p* ≤ 0.05). AS, Artificial Saliva; CT, Colgate Total 12 Clean Mint; CC, Turmeric, Clove and Tea Tree Extracts; CA, Chamomile, Melissa and Grape Extracts; CH, Pepper Mint and Turmeric Extracts;ZM, Zero Mint; ZH, Zero Pepper Mint; ME, Mint Everest; CM, Charcoal and Mint; HA, Herbal Anis Mint and Tea Tree. WAT, acceptability threshold for whiteness differences; WPT, perceptibility threshold for whiteness differences.

Figure 2 depicts the variations in ΔWI_D_. No differences among groups were noted in ΔWI_D_, regardless of the toothbrushing cycles (15.000 or 30.000) (*p* > 0.05). All toothpastes tested exhibited negative values, indicating that none of the groups promoted a whitening effect, and none reached values above 0.7 or 2.6 units, corresponding to perceptibility and acceptability values, respectively.

### 3.2 Percentage of Surface Hardness Loss (%SHL)

**FIGURE 3:**
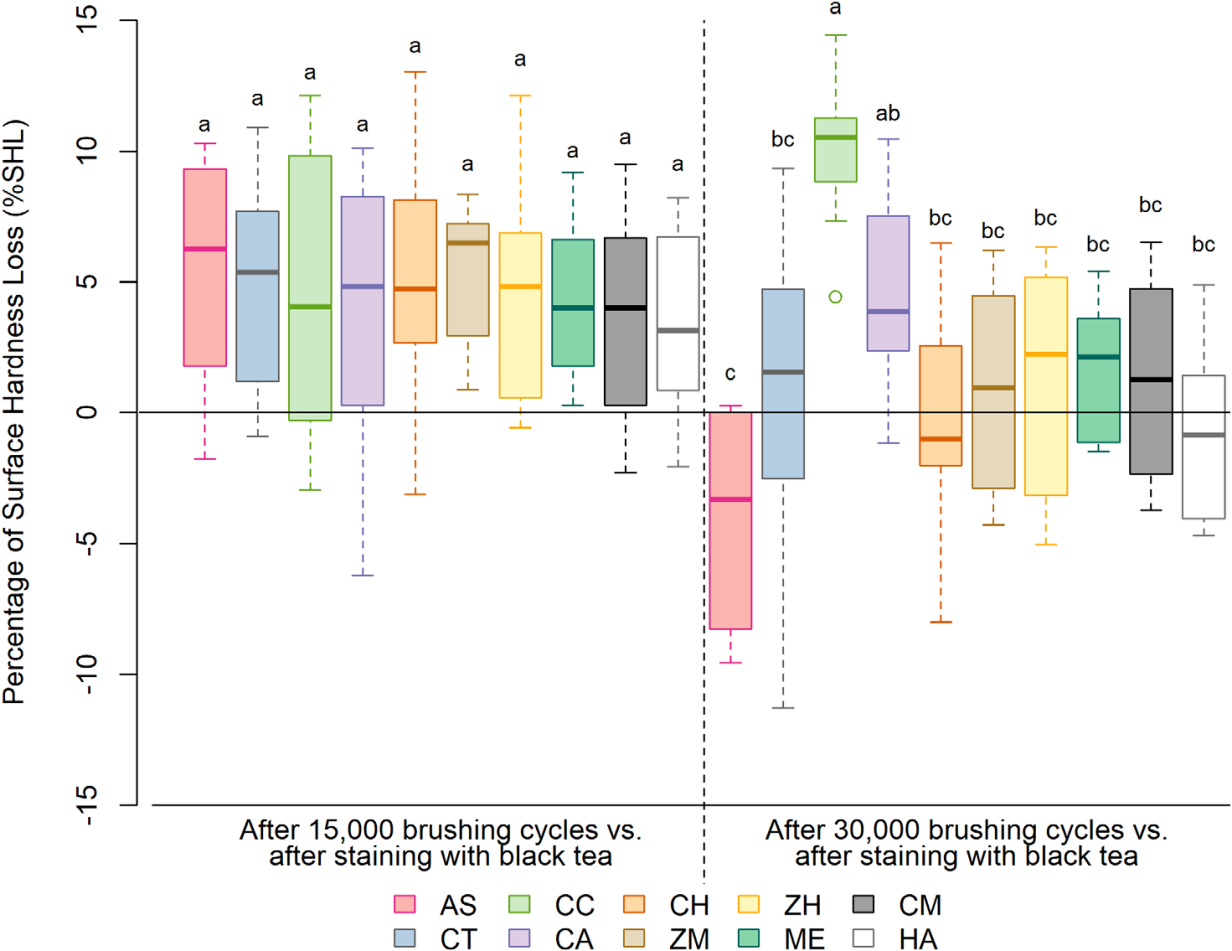
Mean values and standard deviations of %SHL at T1 and T2, respectively. Different letters indicate statistical differences between groups (*p* ≤ 0.05). AS, Artificial Saliva; CT, Colgate Total 12 Clean Mint; CC, Turmeric, Clove and Tea Tree Extracts; CA, Chamomile, Melissa and Grape Extracts; CH, Pepper Mint and Turmeric Extracts; ZM, Zero Mint; ZH, Zero Pepper Mint; ME, Mint Everest; CM, Charcoal and Mint; HA, Herbal Anis Mint and Tea Tree.

Figure 3 shows median values of %SHL. No differences among groups were observed at T1, and toothbrushing for 15.000 cycles did not significantly change enamel surface microhardness (*p* > 0.05). However, after 30.000 cycles, the CC group showed greater hardness loss than the other groups (*p* < 0.05), except for the CA group, which did not differ significantly from CC (*p* > 0.05). Besides, CC and CA showed greater hardness loss than the AS group (*p* < 0.05).

### 3.3 Surface Roughness

**FIGURE 4:**
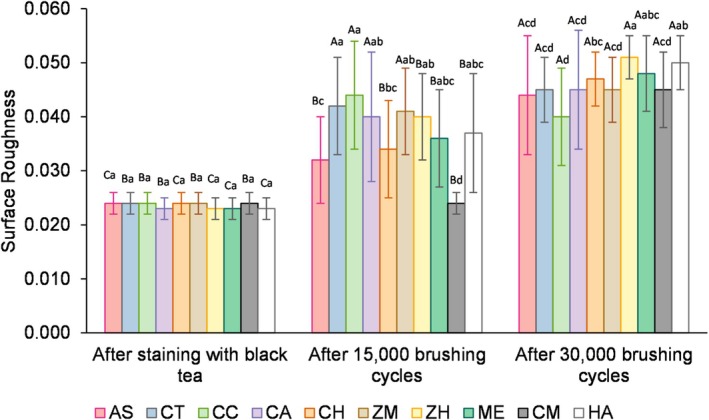
Mean values and standard deviations of surface roughness at T1 and T2, respectively. Different letters indicate statistical differences between groups (*p* ≤ 0.05). AS, Artificial Saliva; CT, Colgate Total 12 Clean Mint; CC, Turmeric, Clove and Tea Tree Extracts; CA, Chamomile, Melissa and Grape Extracts; CH, Pepper Mint and Turmeric Extracts;ZM, Zero Mint; ZH, Zero Pepper Mint; ME, Mint Everest; CM, Charcoal and Mint; HA, Herbal Anis Mint and Tea Tree.

Figure 4 displays the significant interaction found among the factors “toothpastes” (*p* = 0.0227), “time” (*p* < 0.0001), and “toothpastes × time” (*p* = 0.0077). Although staining (T0) did not influence surface roughness (*p* > 0.05) as no differences were found among groups, 15.000 toothbrushing cycles (T1) with the toothpastes tested increased surface roughness (*p* < 0.05), except for the dentifrice CM, which maintained Ra values similar to T0 (*p* < 0.05). Moreover, compared to T1, enamel surface roughness increased following toothbrushing for 30.000 cycles (T2), except for the dentifrice CU, which was similar to T1 (*p* > 0.05). At T1, CM exhibited the lowest Ra values among groups, while at T2, ZH showed higher Ra than most of the groups (*p* < 0.05). At T2, enamel surface roughness of all groups increased compared to T0 (*p* < 0.05).

### 3.4 SEM and EDS Analysis

**FIGURE 5:**
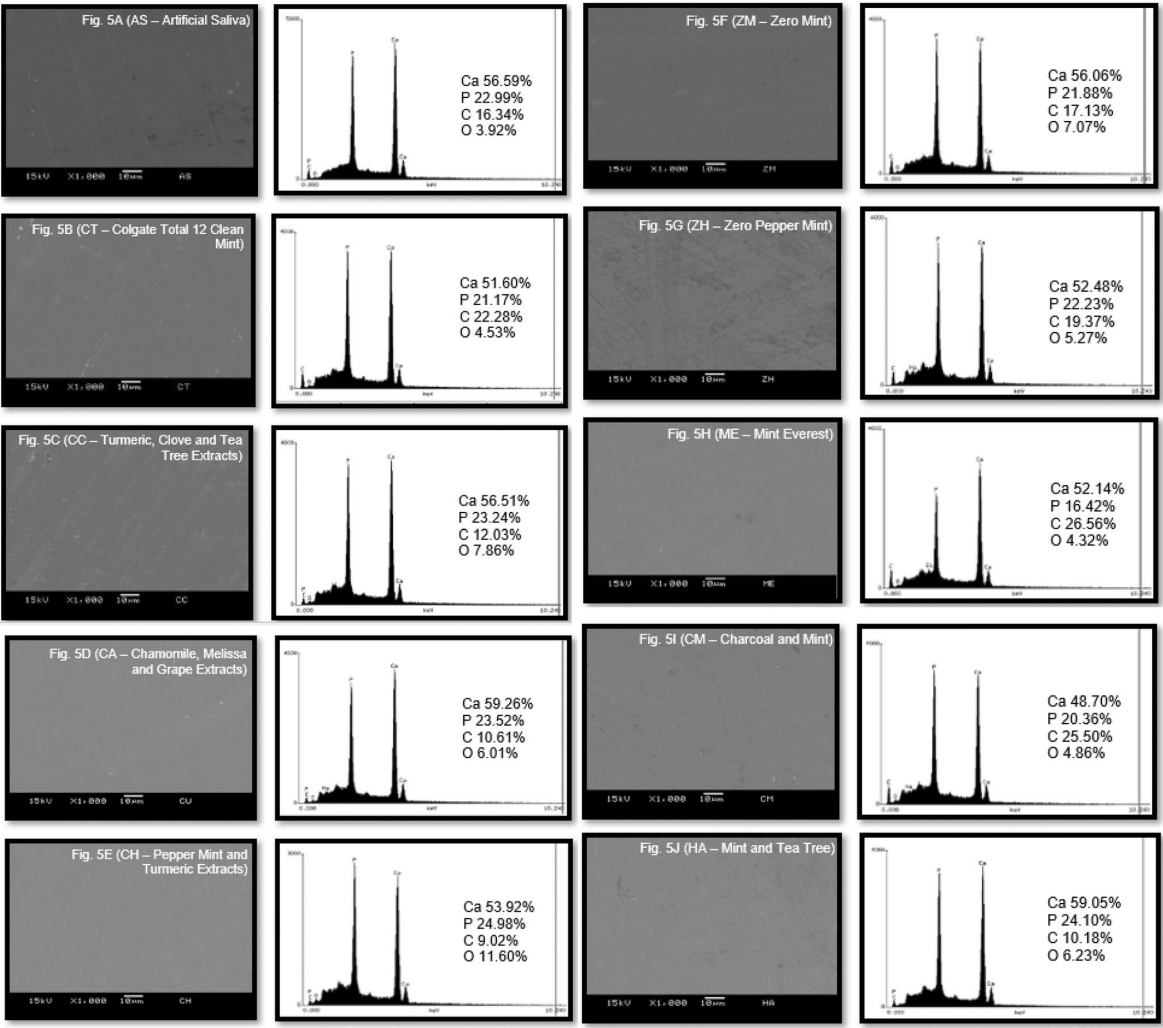
Scanning electron microscopy (SEM) images and energy‐dispersive X‐ray spectroscopy (EDS) after T2. No significant changes in morphology or % weight of chemical elements between groups were observed.

Figure 5 (A–J) shows the mean percentages (%) of atomic weight for mineral content on enamel determined by EDS. No significant variations in mineral content were detected among the groups. Representative SEM images and EDS graphs indicate that toothpastes did not promote a high alteration in the enamel surface at T2.

## 4 Discussion

This study analyzed the ability of toothbrushing with vegan toothpastes to promote a whitening effect and its potential impact on enamel surface hardness, roughness, mineral content, and morphology. Before brushing, the samples were subjected to the artificial staining protocol to simulate tooth discoloration and evaluate vegan toothpastes' ability to remove extrinsic stains. Black tea solution was chosen due to its high staining potential [20], its ability to decrease the luminosity (−L*) and intensify the red (+a*) and yellow (+b*) tones of the surface, and have greater ease of penetration (because of its low molecular weight) [21], since this has been confirmed in the initial analysis after staining with black tea at T0. Our results indicate that the black tea solution notably stained the enamel surface and ZM, ZH, ME, and CM at T2 possibly removed part of the extrinsic stains compared to the other groups.

Although Turmeric and Charcoal were once associated with “natural teeth whitening,” only CM manufacturer states that the product has this purpose. Therefore, to evaluate whether CC, CH (toothpastes with Turmeric and with orange appearance), and CM (toothpaste with Charcoal) provided a whitening effect, the WI_D_ was determined. Positive and high WI_D_ values suggest a greater perception of whitening, while low and negative values are associated with a lower perception of whitening [22, 23]. ΔWI_D_ values lower than 0.7 units are not noticeable to an average observer [6, 23]. According to the results, all groups exhibited negative WI_D_ values, clearly indicating that none of the vegan toothpastes tested promoted a whitening effect, consequently, these products were not able to remove the stains caused by staining of black tea. Most charcoal products are marketed as color‐changing products and are advertised as over‐the‐counter whitening products. A systematic review developed in 2023 [24] found that activated charcoal toothpastes have a lower whitening effect than other alternatives and may be considered less safe due to their high abrasive potential. Thus, although all groups exhibited color change, the first null hypothesis was accepted, because none of them promoted a PT and AT whitening effect.

Surface microhardness analysis evaluated the indirect enamel mineral content after brushing cycles, considering that fluoride in the toothpaste composition (CT, ZM, ZH, ME, HA) or its absence (CC, CA, CH, CM) could affect the enamel hardness. At baseline, enamel hardness was analyzed, and only specimens with close SMH values were selected and randomly distributed into the groups. Thus, groups exhibited no microhardness differences at T0. After T1, none of the toothpastes changed enamel surface microhardness. Only CC exhibited a statistically significant hardness loss following T2. At the same time, CC and CA showed higher %SHL than the control group, which remained in artificial saliva. These results led us to reject the second null hypothesis. However, it should be noted that, generally, the median %SHL of all groups was within approximately 10% of the original hardness value. Thus, the hardness variation could be considered standard and clinically irrelevant to the enamel structure, and we assume that it did not have a critical effect on enamel mineral content.

The %SHL results can also be explained by ingredients such as turmeric and grape seed, present in CC and CA toothpastes, respectively. It is possible that there was partial remineralization of the enamel due to the presence of polyphenols in their composition. Polyphenols are incorporated into the structure of the salivary pellicle, interact and crosslink the existing proteins, thereby increasing the binding of salivary proteins. This process results in possibly increasing a thicker and denser pellicle, which enhances its resistance to acidic attacks [25]. Polyphenolic agents are associated with remineralization and demineralization of dental structures, and the literature well describes fluoride's beneficial effects in this process. It acts as a catalyst in the demineralization and remineralization process, accelerating remineralization by approximately five times. This is possible due to the formation of a reservoir in the saliva, which remains for a few hours, and its aggregation in the dental biofilm, which lasts for a prolonged period [26].

Fluoride can be found in different formulations, such as NaF, Na_2_FPO_3_, C_27_H_60_F_2_N_2_O_3_, SnF_2_, and AmF, or in combination [27]. This study's toothpaste formulations contain sodium fluoride (NaF) and sodium monofluorophosphate (MFP). In the form of MFP, fluoride becomes available when hydrolyzed in the oral cavity by phosphatase enzymes in human saliva [28]. Since artificial saliva was used to simulate the oral cavity, the MFP remineralizing effects may not have been evident under the in vitro conditions simulated in this study.

Furthermore, xylitol (ingredient present in all toothpastes without fluoride) is a natural sweetener that is as sweet as normal sugar (sucrose) and is used as a sugar substitute. Also, it has other properties that help in the prevention of tooth wear, responsible for increasing mineralization on dental tissues. Xylitol has the ability to form complexes with Ca^2+^ ions, facilitating their transport and incorporation into dental lesions, thereby promoting the rehardening of the demineralized enamel layer [29]. The remineralization process occurs due to enhanced saliva flow that is rich in phosphate and calcium [30, 31]. The presence of this ingredient may also have influenced the results. Besides, the inorganic content gain or loss could have been different if the treatments had been under a dynamic pH cycling model [32]. In this context, future evaluations could explore the behavior of vegan toothpaste without fluoride in a more challenging environment, reflecting more closely the oral conditions. Hence, although hardness loss was detected for CC and CA after 30.000 brushing cycles, we assume that these results do not trigger deleterious effects on the microhardness of tooth enamel.

The third null hypothesis was rejected since toothbrushing with vegan toothpastes increased enamel surface roughness (Ra). To standardize the specimens and allow comparison among groups, the enamel surface of all specimens was polished to reach a surface roughness average of 0.02 μm at baseline. The increase in surface roughness, for all groups at the end of T2, can be attributed to the mechanical action and abrasion promoted by brushing with the vegan toothpastes. These results confirm that longer toothbrushing influences the enamel topography [33] mainly because of the contact of the brushing bristles combined with the abrasive components of the toothpastes.

According to the composition displayed by the manufacturer, the toothpastes used herein present hydrated silica, calcium carbonate, sodium bicarbonate, sodium silicate, calcium carbonate, tetrasodium pyrophosphate, disodium pyrophosphate, and one of them displays activated charcoal (CM). Although it was expected that the activated charcoal would exceed the surface roughness in comparison with the other groups, interestingly, after T1, it displayed the lowest Ra values among the groups. However, as previously mentioned, after T2, the behavior of this group was comparable to the remaining toothpastes without activated charcoal.

These results partially disagree with a previous investigation, in which the authors observed surface roughness did not increase after toothbrushing with activated charcoal toothpaste without fluoride [34]. In that study, the authors aimed to evaluate surface roughness (Sa), roughness profile (Rv), and enamel wear after brushing with different whitening toothpastes and charcoal powders, simulating 30.000 brushing cycles. As mentioned, all toothpastes were responsible for increasing Ra after T2. But similar to the microhardness evaluation, the final roughness outcomes are distant from the threshold previously established which attests that only surface roughness above 0.20 μm leads to dental biofilm accumulation [35], and none of the toothpastes exceeded this Ra value. Thus, although groups may present statistical differences that rejected the third hypothesis, none of them provided an outcome that could possibly be deleterious to the enamel surface.

SEM images exhibited the enamel morphology following toothbrushing with vegan toothpastes, while EDS analysis identified the enamel chemical composition. No significant changes were noted on enamel following 30.000 brushing cycles, and the minor surface irregularities observed agree with the roughness findings. Besides, similar mineral content was identified in all groups, corroborating the microhardness findings and leading us to conclude that the vegan toothpastes tested did not promote harmful effects on the enamel surface.

The abrasiveness of toothpastes is closely related to the type and quantity of abrasive agents in the toothpaste composition. To allow for the removal of soft deposits and extrinsic stains, phosphates, carbonates, and silicas have been frequently used in commercial toothpastes [36]. However, the abrasive capacity of toothpastes must be adequate to perform efficient cleaning without damaging the integrity of the tooth structure. The literature reports a classification of toothpastes according to their RDA, which must be equal to or less than 250, as these values are considered safe and effective [37]. Colgate Total 12 Clean Mint, used as a conventional toothpaste, presents an RDA of 139.12 [38], and although not all of the toothpastes used display the RDA values, most manufacturers do not provide these values [39].

Toothpastes with different characteristics and compositions have been suggested for different clinical scenarios, such as anti‐erosion, anti‐caries, periodontal disease control, post‐surgical care, daily oral care, whitening, and vegan habit. However, information regarding the abrasiveness of commercial toothpastes is not always available on the packaging to allow an appropriate choice based on professional recommendations. In general, toothpastes with whitening proposals based on hydrated silica have demonstrated greater abrasive potential than conventional toothpastes [40], but none of the toothpastes tested herein exhibited this tendency.

The main objective of this in vitro research was to analyze whether vegan toothpastes could provide a whitening effect. As expected, none of the products tested provided a whitening effect, as none present a compound capable of breaking down the chromophores, such as hydrogen peroxide. Although the vegan natural compounds might exhibit some degree of whitening, their concentration and the time of contact with the tooth surface were insufficient to provide any color alteration in the brushing simulation protocol performed (15.000 and 30.000 brushing cycles, corresponding to 18 and 36 months of clinical brushing) [13, 14, 15].

Still, another possibility of pigment removal could have been caused by toothpaste abrasion. However, since the tested toothpastes did not present high abrasiveness, pigment removal did not occur. On the one hand, this fact denotes the safe use of the product, as there was no change in roughness or morphology. Nevertheless, none of the toothpastes promote changes in mineral content, which are assumed to be beneficial.

However, it should be noted that in this study, pH cycling was not performed, and therefore, the remineralization capacity of toothpastes, especially those without fluoride, was not evaluated. Thus, we discourage the use of toothpastes without fluoride until further analyses are performed. The lack of dynamic pH cycling can be considered a limitation, and future investigations must simulate the pH changes that occur in oral conditions. Furthermore, profilometric analysis must be used to determine the mineral volume loss in models that simulate pH cycling, in which significant and detectable changes in the enamel surface are expected.

## 5 Conclusions

Within the limitations of this study, it can be concluded that the vegan toothpastes:

– Did not produce a whitening effect;
– Did not significantly alter enamel surface microhardness;
– Increased surface roughness after 30.000 cycles;
– Did not induce morphological changes in the enamel surface.

## Conflicts of Interest

The authors declare no conflicts of interest.

## Data Availability

The data that support the findings of this study are available from the corresponding author upon reasonable request.
